# Erk5 Is a Key Regulator of Naive-Primed Transition and Embryonic Stem Cell Identity

**DOI:** 10.1016/j.celrep.2016.07.033

**Published:** 2016-08-04

**Authors:** Charles A.C. Williams, Rosalia Fernandez-Alonso, Jinhua Wang, Rachel Toth, Nathanael S. Gray, Greg M. Findlay

**Affiliations:** 1 The MRC Protein Phosphorylation and Ubiquitylation Unit, School of Life Sciences, University of Dundee, Dundee DD1 5EH, UK; 2 Department of Cancer Biology, Dana-Farber Cancer Institute, Boston, MA 02215, USA; 3 Department of Biological Chemistry and Molecular Pharmacology, Harvard Medical School, Boston, MA 02115, USA; 4 The Division of Signal Transduction Therapy, School of Life Sciences, University of Dundee, Dundee DD1 5EH, UK

## Abstract

Embryonic stem cells (ESCs) can self-renew or differentiate into any cell type, a phenomenon known as pluripotency. Distinct pluripotent states, termed naive and primed pluripotency, have been described. However, the mechanisms that control naive-primed pluripotent transition are poorly understood. Here, we perform a targeted screen for kinase inhibitors, which modulate the naive-primed pluripotent transition. We find that XMD compounds, which selectively inhibit Erk5 kinase and BET bromodomain family proteins, drive ESCs toward primed pluripotency. Using compound selectivity engineering and CRISPR/Cas9 genome editing, we reveal distinct functions for Erk5 and Brd4 in pluripotency regulation. We show that Erk5 signaling maintains ESCs in the naive state and suppresses progression toward primed pluripotency and neuroectoderm differentiation. Additionally, we identify a specialized role for Erk5 in defining ESC lineage selection, whereby Erk5 inhibits a cardiomyocyte-specific differentiation program. Our data therefore reveal multiple critical functions for Erk5 in controlling ESC identity.

## Highlights

• A kinase inhibitor screen identifies Erk5 as a key regulatory of ESC pluripotency
• Erk5 suppresses transition to primed pluripotency and neural differentiation
• Erk5 controls ESC identity by suppressing cardiomyocyte differentiation

## Graphical Abstract

**Figure:**
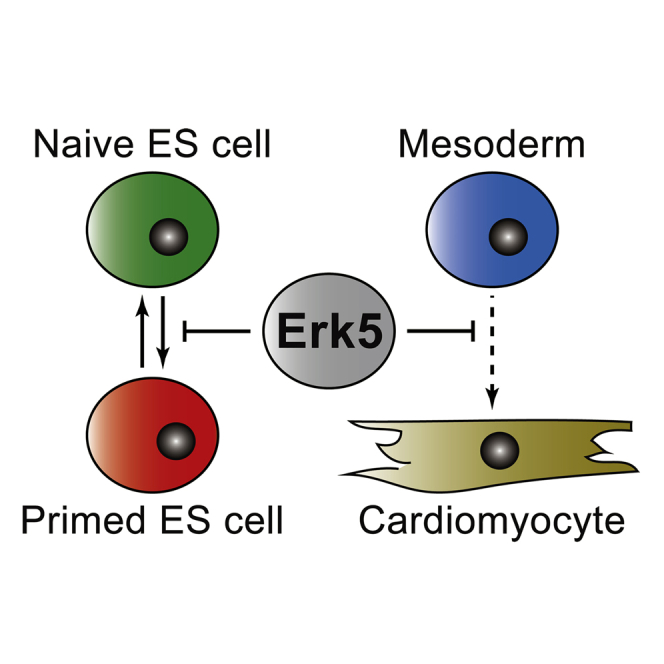

## Teaser

Williams et al. combine chemical screening and genetic approaches to identify Erk5 kinase as a critical regulator of the naive-primed pluripotent transition and cardiomyocyte differentiation.

## Introduction

**Figure 1:**
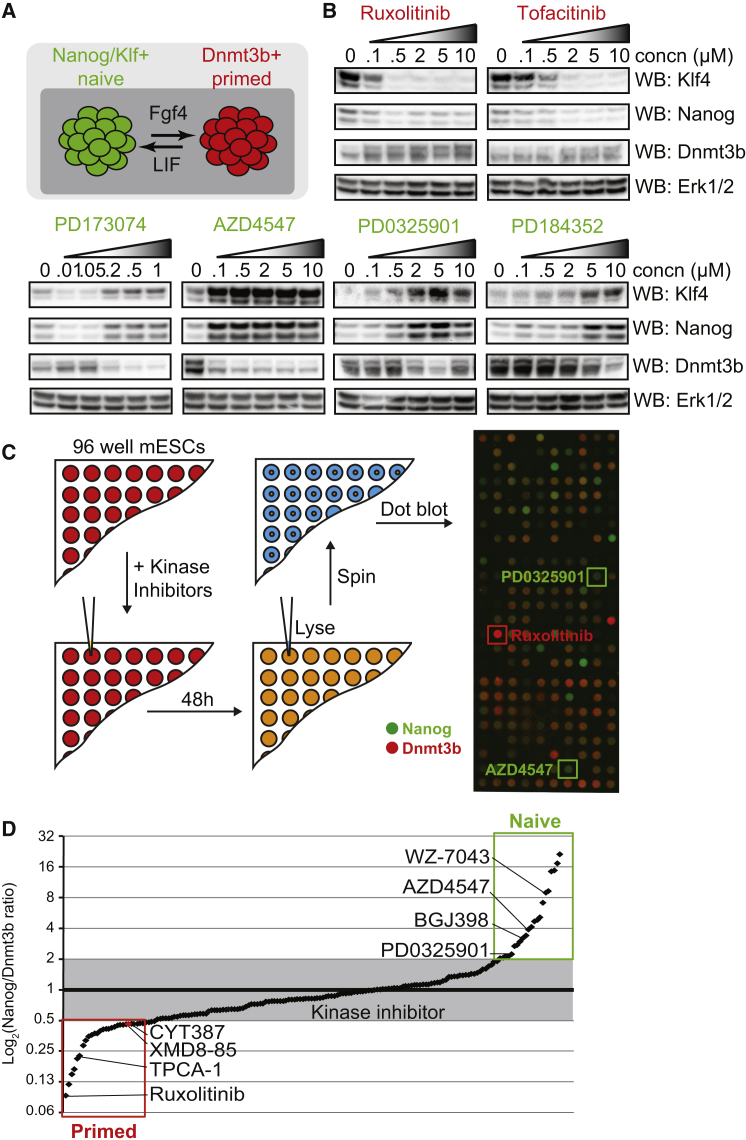
Systematic Identification of Kinase Inhibitors that Modulate Naive-Primed Pluripotent Transition (A) mESCs cultured in LIF/FBS transitioning between naive (green) and primed (red) pluripotent states. (B) mESCs were treated with the indicated concentrations of Jak inhibitors (ruxolitinib and tofacitinib), Fgfr inhibitors (PD173074/AZD4547), or Mek1/2 inhibitors (PD0325901/PD184352). Klf4, Nanog, Dnmt3b, and Erk1/2 levels were determined by immunoblotting. (C) 228 potent and selective kinase inhibitors were screened at 1 μM for effects on pluripotency signature. Nanog and Dnmt3b expression was determined for each inhibitor and images overlaid. Selected positive control inhibitors are highlighted. (D) The Nanog:Dnmt3b ratio for each kinase inhibitor was determined and inhibitors ranked accordingly. Inhibitors found to alter Nanog:Dnmt3b beyond a 2-fold threshold were identified as drivers of naive or primed pluripotency. Selected positive control inhibitors are highlighted. See also Tables S1 and S2.

Embryonic stem cells (ESCs) can self-renew or potentially differentiate into all cell types, a phenomenon known as pluripotency (Evans and Kaufman, 1981). Distinct pluripotent states, termed naive and primed pluripotency, have been described (Nichols and Smith, 2009). Naive ESCs occupy a developmental ground state characteristic of the preimplantation embryo (Boroviak et al., 2015), while primed pluripotent cells resemble post-implantation embryonic epiblast poised for further differentiation (Tesar et al., 2007). Naive pluripotency is marked by expression of key self-renewal factors such as Nanog, Krueppel-like transcription factors (Klfs), Rex1 (Nichols and Smith, 2009), and Esrrb (Festuccia et al., 2012). Conversely, primed pluripotency is characterized by expression of the de novo DNA methyltransferase Dnmt3b (Figure 1A) (Ficz et al., 2011), the epiblast marker Fgf5, and lineage priming factor Brachyury (Nichols and Smith, 2009).

Understanding the mechanisms that control naive-primed pluripotent transitions is fundamental to ESC biology. In this regard, mouse ESCs (mESCs) provide a tractable model, as they undergo dynamic transition between naive and primed pluripotency when cultured in leukemia inhibitory factor (LIF) and fetal bovine serum (FBS) (Chambers et al., 2007, Findlay et al., 2013). LIF-Jak-Stat3 signaling drives expression of naive pluripotency genes (Niwa et al., 1998), while autocrine fibroblast growth factor 4 (Fgf4)-Erk1/2 signaling promotes primed transition (Kunath et al., 2007). However, beyond these and several other core pluripotency pathways, the role of protein kinases in pluripotency regulation has not been systematically evaluated.

Small-molecule screening represents a simple approach to elucidate kinase regulators of pluripotency. In a screen for modifiers of the naive-primed transition, we uncover XMD series compounds, which selectively inhibit the Erk5 kinase and BET bromodomain family, as drivers of primed pluripotency. Using rational inhibitor engineering and genome editing, we deconvolve individual roles of Erk5 and Brd4 in pluripotency regulation. Erk5 promotes expression of a network of naive pluripotency factors, which requires kinase activity, upstream activation by Mek5, and a C-terminal transcriptional domain. Furthermore, Erk5 signaling potently suppresses the transition of naive cells toward primed pluripotency and neuroectoderm differentiation. Finally, we show that Erk5 has a distinct function in suppressing late-stage cardiac gene expression and cardiomyocyte development.

## Results

### A Screen for Kinase Inhibitors that Modulate the Naive-Primed Pluripotent Transition

In order to systematically explore signaling pathways that control the naive-primed transition, we developed a quantitative pluripotency assay based on the naive and primed markers Nanog and Dnmt3b, respectively (Figure 1A). Control inhibitors stabilize naive and primed pluripotent states as expected; LIF-Jak-Stat3 inhibition by ruxolitinib and tofacitinib promotes a primed signature (low Nanog, high Dnmt3b; Figure 1B), while the Fgfr inhibitors PD173074 and AZD4547 or the Mek1/2 inhibitors PD0325901 and PD184352 promote a naive signature (high Nanog, low Dnmt3b; Figure 1B). We therefore exploited this assay to interrogate a targeted collection of 228 potent and selective kinase inhibitors (http://lincs.hms.harvard.edu) and identified those that modulate the naive-primed transition (Figure 1C). Kinase inhibitors that stabilize naive and primed states include many known pluripotency regulators and non-selective compounds (Figure 1D; Tables S1 and S2). However, we prioritized XMD8-85, which promotes primed pluripotency, for follow-up analysis.

### Erk5 and Brd4/BET Have Distinct Functions in Regulating the Naive-Primed Transition

**Figure 2:**
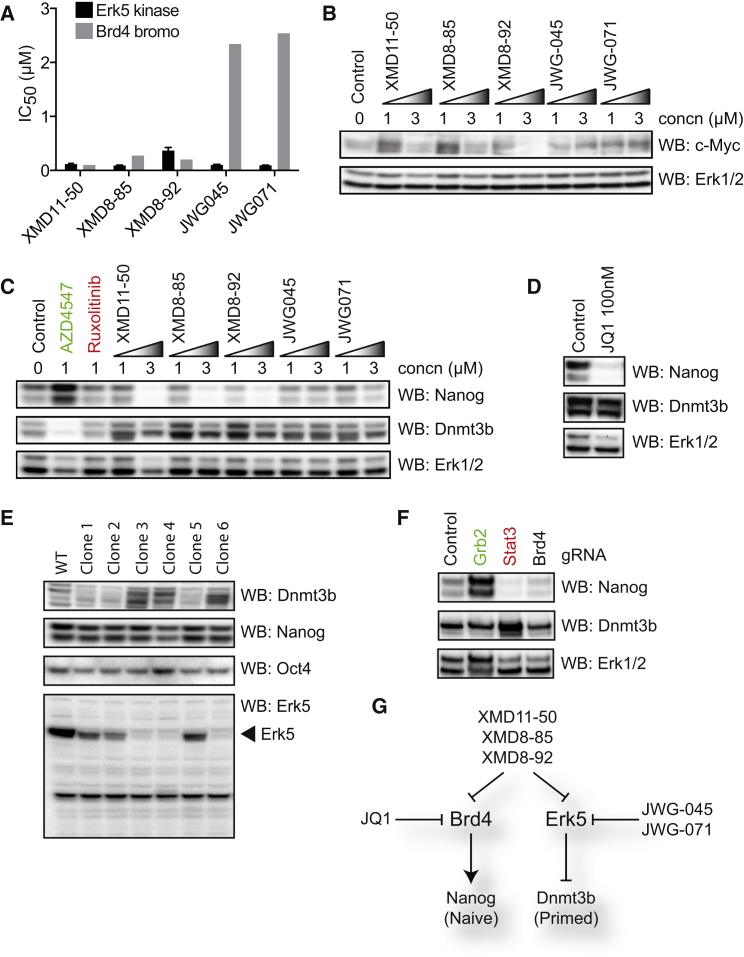
Deconvolution of Distinct Functions for Erk5 and Brd4 in Pluripotency (A) IC_50_ determination for inhibition of Erk5 and Brd4 by XMD and JWG compounds. (B) mESCs were treated with the indicated concentrations of XMD and JWG inhibitors, and c-Myc and Erk1/2 levels were determined by immunoblotting (n = 3). (C) mESCs were treated with 1 μM AZD4547 or ruxolitinib or the indicated concentrations of XMD and JWG inhibitors. Nanog, Dnmt3b, and Erk1/2 levels were then determined by immunoblotting (n = 3). (D) mESCs were treated with 100 nM JQ1 and Nanog, Dnmt3b, and Erk1/2 levels determined by immunoblotting (n = 3). (E) Erk5 gene targeted mESC clones were generated using CRISPR/Cas9 D10A. Dnmt3b, Nanog, Oct4, and Erk5 levels were then determined by immunoblotting (n = 3). (F) mESCs were transiently transfected with Cas9 D10A and either control or gRNAs targeting Grb2, Stat3, or Brd4. Nanog, Dnmt3b, and Erk1/2 levels were determined by immunoblotting (n = 3). (G) Deconvolution of the role of Erk5 and Brd4 in pluripotency regulation. See also Figure S1 and Table S3.

Among kinases, XMD8-85 and related compounds are selective Erk5 inhibitors (Deng et al., 2011) but also inhibit Brd4/BET family bromodomains, transcriptional regulators required for Nanog expression (Di Micco et al., 2014, Horne et al., 2015, Liu et al., 2014). This could potentially account for the primed pluripotent phenotype obtained with XMD8-85, which prompted us to deconvolute the individual functions of Erk5 and Brd4/BET during the naive-primed transition. Thus, we rationally engineered two compounds with reduced Brd4/BET inhibitory activity, JWG-045 and JWG-071. In contrast to XMD, which displays relatively high affinity for Brd4, JWG has significantly reduced Brd4 affinity but comparable Erk5 affinity (Figure 2A). Accordingly, JWG does not suppress the Brd4 target gene c-Myc, unlike XMD (Figure 2B). Interestingly, lower concentrations of either compound series stabilizes c-Myc, which is explained by inhibition of Erk5-dependent c-Myc phosphorylation and degradation (English et al., 1998).

We then compared the effects of XMD and JWG on the Nanog/Dnmt3b pluripotency signature. XMD compounds suppress Nanog expression (Figure 2C; Figure S1A), consistent with the role of Brd4 in Nanog regulation (Di Micco et al., 2014, Horne et al., 2015, Liu et al., 2014). XMD treatment also promotes Dnmt3b expression (Figure 2C; Figure S1A), although this is reduced at 3 μM (Figure 2C) due to loss of cell viability (Figure S1B). In contrast, JWG compounds engineered for reduced Brd4/BET inhibition do not alter Nanog expression (Figure 2C; Figure S1A). However, Dnmt3b protein (Figure 2C; Figure S1A) or mRNA (Figure S1C) expression is elevated following either XMD or JWG treatment, suggesting that specific Erk5 inhibition drives Dnmt3b expression. Congruently, the BET inhibitor JQ1, which does not inhibit Erk5 (Malik et al., 2015), suppresses Nanog without Dnmt3b induction (Figure 2D), indicating that XMD compounds modulate the naive-primed transition via inhibition of both Erk5 and Brd4/BET.

### Genome Editing Confirms that Erk5 Functions in Pluripotency Regulation

Small molecules frequently exert cryptic off-target effects (Bain et al., 2007), which prompted us to use CRISPR/Cas9 D10A paired with tandem gRNAs (Ran et al., 2013) to selectively disrupt the *Mapk7* (Erk5) and *Brd4* genes. Multiple Erk5^−/−^ mESC clones display elevated Dnmt3b expression, while Nanog remains relatively unperturbed (Figure 2E; Figure S1C), corroborating our inhibitor data. Importantly, Erk5 knockout does not influence the pluripotency master regulator Oct4 (Figure 2E). We were unable to isolate stable Brd4^−/−^ mESC clones (Di Micco et al., 2014), but we conducted targeted gene disruption by transient transfection. Knockout of the key LIF and Fgf signaling components Stat3 and Grb2 promotes primed and naive pluripotency respectively (Figure 2F), providing proof of principle for this approach. Brd4 knockout suppresses Nanog expression without altering Dnmt3b (Figure 2F), consistent with effects of the Brd4/BET inhibitor JQ1 (Figure 2C). We confirm that expression of Grb2, Stat3, and Brd4 is efficiently disrupted (Figure S1D). In summary, we provide multiple lines of evidence that Erk5 suppresses Dnmt3b and the transition to primed pluripotency (Figure 2G).

### Erk5 Promotes a Naive Pluripotency Network to Suppresses ESC Priming

**Figure 3:**
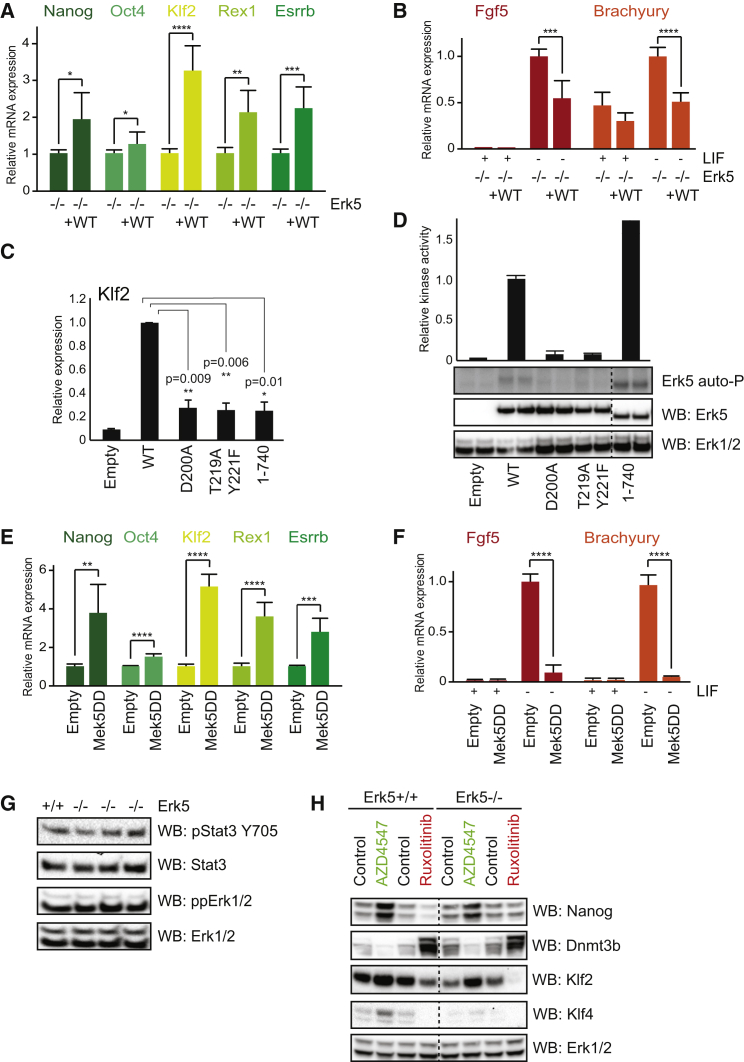
Erk5 Signaling Stabilizes Naive Pluripotency and Suppresses Transition to Primed (A) Erk5^−/−^ mESCs were transfected with empty vector or Erk5 cDNA and Nanog, Oct4, Klf2, Rex1, and Esrrb mRNA levels determined by qRT-PCR following 3-day LIF withdrawal. Data represent average ± SD (n = 3). (B) Erk5^−/−^ mESCs were transfected with empty vector or Erk5 cDNA. Fgf5 and Brachyury mRNA levels were determined by qRT-PCR following 3 days in the presence or absence of LIF. Data represent average ± SD (n = 3). (C) Erk5^−/−^ mESCs were transfected with empty vector or Erk5 constructs and Klf2 protein expression determined by immunoblotting and normalized. Data are presented as average ± SD (n = 3). (D) Erk5^−/−^ mESCs were transfected with empty vector or Erk5 constructs and stimulated with H_2_O_2_, and Erk5 kinase activity was determined. Erk5 and Erk1/2 expression levels were determined by immunoblotting. Data represent average ± SD (n = 3). Intervening lanes were removed, indicated by a dotted line. (E) Erk5^+/+^ mESCs were transfected with empty vector or Mek5DD cDNA. Nanog, Oct4, Klf2, Rex1, and Esrrb mRNA levels were then determined by qRT-PCR following 3 days of LIF withdrawal. Data represent average ± SD (n = 3). (F) Erk5^+/+^ mESCs were transfected with empty vector or Mek5DD, and Fgf5 and Brachyury mRNA levels were determined by qRT-PCR after 4 or 5 days in the presence or absence of LIF, respectively. Data represent average ± SD (n = 3). (G) Stat3 pTyr705, total Stat3, Erk1/2 pThr202/Tyr204, and total Erk1/2 levels in Erk5^+/+^ or Erk5^−/−^ mESC clones were determined by immunoblotting (n = 3). (H) Erk5^+/+^ and Erk5^−/−^ mESCs were treated with 1 μM AZD4547 or ruxolitinib. Nanog, Dnmt3b, Klf2, Klf4, and Erk1/2 levels were then determined by immunoblotting. Intervening lanes were removed, as indicated by a dotted line (n = 3). See also Figure S2.

Our findings prompted us to examine the influence of Erk5 on the extended pluripotency network. Employing Erk5^−/−^ mESCs re-expressing Erk5 at endogenous levels (Figure S2A), we find that Erk5 modestly influences Oct4, while Nanog is subtly but significantly induced by Erk5 (Figure 3A). However, Erk5 robustly maintains expression of key naive pluripotency factors Klf2, Rex1, and Esrrb following LIF withdrawal (Figure 3A; Figure S2A), suggesting that Erk5 maintains the naive state even under conditions favoring the transition toward primed pluripotency. We tested this directly by examining the primed pluripotency markers Fgf5 and Brachyury following LIF withdrawal (Figure 3B). Fgf5 and Brachyury induction is significantly suppressed by Erk5 (Figure 3B), confirming that Erk5 functions to restrain the naive-primed transition by a range of molecular criteria.

### Erk5 Kinase Activity and Transcriptional Domain Are Required to Maintain Naive Pluripotency

Erk5 comprises a kinase domain that is phosphorylated and activated by Mek5, C-terminal autophosphorylation sites, and a transcriptional activation domain (Akaike et al., 2004). Erk5 expression in Erk5^−/−^ mESCs promotes Klf2 induction upon LIF withdrawal, which requires Erk5 kinase activity (D200A) and upstream phosphorylation by Mek5 (T219A Y221F; Figure 3C). The Mek5 inhibitor BIX02189 also suppresses Klf2 expression in wild-type mESCs (Figure S2B). Interestingly, a truncation that disrupts Erk5 transcriptional activity (1–740) similarly suppresses Klf2 induction. We assessed the effect of these mutations on Erk5 kinase activity (Morimoto et al., 2007), which is abolished by mutating the catalytic aspartate (D200A) or the activation loop motif phosphorylated by Mek5 (T219A Y221F; Figure 3D). However, truncation of the transcriptional domain (1–740) does not suppress Erk5 kinase activity (Figure 3D) but fails to rescue Klf2 expression in Erk5^−/−^ mESCs (Figure 3C), indicating that Erk5 kinase activity and transcriptional activity are critical for the maintenance of naive pluripotency.

### Mek5 Signaling to Erk5 Potently Stabilizes the Naive State to Block the Transition to Primed Pluripotency

We hypothesized that constitutive Erk5 activation is sufficient to robustly stabilize naive pluripotency and suppress a transition to the primed state. To explore this possibility, we exploited a constitutively activate Mek5 mutant, Mek5 S313D/T317D (hereafter Mek5DD). Mek5DD expression has relatively minor effects on Oct4 but robustly maintains key naive markers Nanog, Klf2, Esrrb, and Rex1 following LIF withdrawal (Figure 3E), indicating that Erk5 pathway activation promotes the naive state. Thus, we tested whether Mek5DD suppresses the transition of mESCs toward the primed state. Fgf5 and Brachyury induction following LIF withdrawal is robustly suppressed by Mek5DD expression to a level approaching that observed in mESCs cultured in LIF (Figure 3F). In addition, we find that Mek5DD expression upon LIF withdrawal delays Fgf5 induction (Figure S2C), indicating that Erk5 pathway activation alters kinetics of the naive-primed transition. Our data therefore provide compelling evidence that Erk5 signaling modulates pluripotency genes so as to inhibit and/or delay the transition of ESCs from naive pluripotency to the primed state.

### Erk5 Functions in Parallel with LIF/FGF Signaling

We then asked whether Erk5 modulates the prominent pluripotency pathways LIF and Fgf (Figure 1B). Analysis of Stat3 and Erk1/2 phosphorylation indicates that Erk5 does not directly modulate LIF or Fgf signaling (Figure 3G). Furthermore, Jak and Fgfr inhibitors promote primed and naive pluripotency signatures in Erk5^+/+^ or Erk5^−/−^ mESCs (Figure 3H), confirming that Erk5 does not modulate transcriptional responses to these key pathways. Indeed, endogenous Erk5 does not prevent loss of Klf4 expression following LIF deprivation (Figure S2D), although Erk5 overexpression in Erk5^−/−^ mESCs can maintain Klf4 expression following LIF withdrawal (Figure S2E). Therefore, although Erk5 modulates Klf4, it does not directly impact LIF signaling.

### Erk5 Maintains Naive Pluripotent ESC Morphology and Restrains Neuroectoderm Differentiation

**Figure 4:**
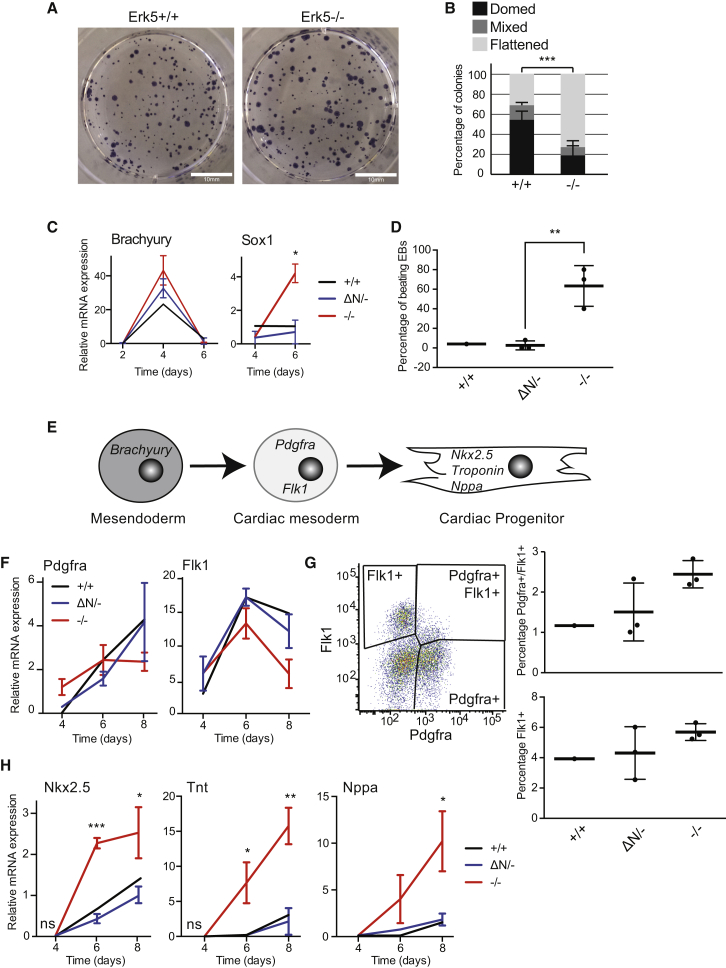
Erk5 Controls Neuroectoderm and Cardiomyocyte Specification of Differentiating ESCs (A) Alkaline phosphatase staining of Erk5^+/+^ and Erk5^−/−^ mESC colonies. (B) Analysis of colony morphology of Erk5^+/+^ and Erk5^−/−^ mESC colonies. Data represent average ± SD (n = 3). (C) Relative mRNA expression of Brachyury and Sox1 were determined for Erk5^+/+^, Erk5^ΔN/−^ (three independent clones) and Erk5^−/−^ (three independent clones) mESCs. Data represent the average of all clones ± SD from a representative experiment (n = 3). (D) Percentage of EBs displaying beating areas derived from Erk5^+/+^, Erk5^ΔN/−^, and Erk5^−/−^ mESCs. Data represent the average of all clones ± SD from a representative experiment (n = 3). (E) Scheme outlining stages of cardiac differentiation. (F) Relative mRNA expression of Pdgfra and Flk1 was determined for Erk5^+/+^, Erk5^ΔN/−^, and Erk5^−/−^ mESCs. Data represent the average of all clones ± SD from a representative experiment (n = 3). (G) FACS quantification of Pdgfra+/Flk1+ cardiac and Flk1+ endothelial progenitors recovered from each cell line. A representative FACS plot illustrating the distinct populations is provided. Data represent average ± SD from a representative experiment (n = 3). (H) mRNA expression levels of Nkx2.5, Tnt, and Nppa were determined for Erk5^+/+^, Erk5^ΔN/−^, and Erk5^−/−^ mESCs. Data represent the average of all clones ± SD from a representative experiment (n = 2). See also Figure S3.

Our demonstration that Erk5 drives a naive pluripotent signature prompted us to examine Erk5^−/−^ mESC morphology. Erk5^−/−^ mESC colonies stain positive for alkaline phosphatase (AP; Figure 4A), confirming that these cells are pluripotent. Analysis of mESC colony morphology indicates that Erk5^+/+^ mESCs prevalently display a “domed,” naive morphology, while Erk5^−/−^ mESC lines primarily form flattened, primed colonies (Figure 4B; Figure S3A). Importantly, Erk5 does not affect the proliferation or survival of mESCs (Figure S3B).

We next examined whether Erk5 controls pluripotent exit and ESC differentiation. Culture of ESCs as embryoid bodies (EBs) mimics differentiation of pluripotent cells during development. We employed a panel of mESC lines generated by CRISPR/Cas9 (control Erk5^+/+^ mESCs, three independent heterozygous Erk5 clones expressing a short N-terminally truncated Erk5 [Erk5^ΔN/−^], and three independent Erk5^−/−^ mESC clones; Table S3) and examined molecular markers of two major differentiation pathways: Brachyury, a mesendoderm marker, and Sox1, a marker of neuroectoderm. Sox1 is significantly increased in Erk5^−/−^ EBs (Figure 4C), suggesting that Erk5 suppresses ESC differentiation to neuroectoderm. In contrast, Brachyury induction in EBs is unaffected by Erk5 status (Figure 4C), suggesting that Erk5 specifically controls pluripotent exit toward the neuroectoderm lineage.

### Erk5 Controls Cardiomyocyte Differentiation Independent of Pluripotency Regulation

Developmental genetics indicate that Erk5 plays a key role in cardiovascular development (Sohn et al., 2002, Yan et al., 2003), which prompted us to examine the role of Erk5 in cardiomyocyte differentiation. Remarkably, we observe a significantly increased percentage of Erk5^−/−^ EBs displaying a “beating” phenotype compared to EBs expressing Erk5 (Figure 4D) or Erk5^−/−^ EBs re-expressing Erk5 (Figure S3C). We addressed the specific developmental stage(s) at which Erk5 influences cardiomyocyte differentiation (Figure 4E). Interestingly, Erk5 does not influence EB differentiation to Brachyury+ mesendoderm (Figure 4C) or induction of the cardiovascular progenitor markers Pdgfra and Flk1 (Figure 4F), suggesting that Erk5 function during cardiomyocyte differentiation is distinct from its role in pluripotency regulation. Fluorescence-activated cell sorting (FACS)-based quantification of cardiovascular progenitor populations reveals a subtle increase in specification of Pdgfra+/Flk1+ cardiac and Pdgfra−/Flk1+ endothelial precursors in Erk5^−/−^ EBs (Figure 4G), Accordingly, Erk5^−/−^ EBs significantly increase the expression of late-stage cardiac-specific genes, including the master regulator Nkx2.5 (Tanaka et al., 1999) and the key cardiac physiology genes troponin T (Tnt) and natriuretic peptide a (Nppa) (Figure 4H). EB morphology is similar between Erk5^+/+^ and Erk5^−/−^ mESCs (Figure S3D). Our data therefore identify a key function for Erk5 in restricting cardiac-specific gene expression and cardiomyocyte differentiation, which is distinct from both the cardiovascular phenotype observed in Erk5^−/−^ mice (Regan et al., 2002, Yan et al., 2003) and Erk5 function in regulating naive-primed transition in ESCs.

## Discussion

In this study, we develop a small-molecule screen and identify XMD8-85 as a driver of the naive-primed transition. XMD series compounds inhibit Erk5 kinase and BET bromodomain family, and we use rational engineering to deconvolute the function of Erk5 in pluripotency regulation. Orthogonal confirmation using CRISPR/Cas9 genome editing technology provides compelling evidence that Erk5 is a key regulator of pluripotency. We propose that this workflow presents a robust and adaptable method to confirm novel targets identified by small-molecule screens in biological systems.

Erk5 kinase activity promotes a key network of naive pluripotency factors, including Klf2, Essrb, and Rex1, which suppresses the transition of naive cells toward primed pluripotency. Interestingly, a C-terminal region of Erk5 not required for kinase activity is essential to maintain naive pluripotency. Phosphorylation of this region drives nuclear localization (Díaz-Rodríguez and Pandiella, 2010, Iñesta-Vaquera et al., 2010) and transcriptional activation in concert with the Mef2- and/or Sp-family transcription factors (Kato et al., 1997, Sunadome et al., 2011, Yan et al., 2001), suggesting a mechanism by which Erk5 promotes naive pluripotency. Intriguingly, Sp1 and Mef2 transcription factors function to modulate Klf expression downstream of Erk5 (Morikawa et al., 2016, Parmar et al., 2006, Sunadome et al., 2011).

We also reveal that Erk5 plays a role in cardiac specification, which was not previously appreciated from in vivo studies (Sohn et al., 2002, Yan et al., 2003). Failure to elaborate proper vasculature around the heart causes the lethality observed in Erk5^−/−^ mice, suggesting that Erk5 may function as a developmental switch to ensure cardiovascular cell types are appropriately specified. Our data therefore argue that Erk5 has independent functions in maintaining naive pluripotency and controlling lineage allocation of differentiating cells. Future investigations will focus on identifying the apparently distinct mechanisms by which Erk5 controls pluripotency and cardiovascular development. Furthermore, our data suggest that Erk5 activators and small-molecule inhibitors are useful tools to modulate cell fate during regenerative approaches such as somatic cell reprogramming and directed differentiation.

## Experimental Procedures

Many reagents generated for this study are available by request at the MRC-PPU reagents website (https://mrcppureagents.dundee.ac.uk/).

### Antibodies and Chemicals

Antibodies used were Nanog (ReproCell Inc.), Dnmt3b (Imgenex), Klf4 (R&D Systems), Erk1/2 and Oct4 (Santa Cruz Biotechnology), phospho-p44/42 MAPK (Erk1/2 Thr202/Tyr204), Stat3α, phospho-Stat3 (Tyr705) and c-Myc (Cell Signaling Technology), Erk5 (Division of Signal Transduction Therapy, Dundee, UK), Klf2 (Millipore), CD309 (Flk1) APC, Clone Avas 12a1, and CD140a (PDGFRα) PE, Clone APA5 (eBioscience). AZD4547, PD173074, PD0325901, PD184352, ruxolitinib, and tofacitinib were from the DSTT (Dundee, UK). The 228 kinase inhibitor library was curated by the Gray lab (http://lincs.hms.harvard.edu). JWG-071 and JWG-045 were synthesized by the Gray lab.

### mESC Culture, Transfection, and Lysis

mESCs were cultured on gelatin coated plates in media containing LIF, 10% fetal calf serum (Gibco), and 5% knockout serum replacement (Invitrogen) unless otherwise stated. mESCs cells were transfected using Lipofectamine LTX (Life Technologies) and selected with puromycin for 48 hr. For CRISPR/Cas9, mESCs were transfected with pX335 and pKN7 (Addgene) and selected, then either lysed or clones isolated. To generate stable lines, Erk5−/− mESCs were electroporated with 30 μg linearized pCAGGS vector, plated at clonal density, and clones were analyzed by immunoblotting. Cell extracts were made in lysis buffer (20 mM Tris [pH 7.4], 150 mM NaCl, 1 mM EDTA, 1% NP-40 [v/v], 0.5% sodium deoxycholate [w/v], 10 mM β-glycerophosphate, 10 mM sodium pyrophosphate, 1 mM NaF, 2 mM Na_3_VO_4_, and Roche Complete Protease Inhibitor Cocktail Tablets).

### Nanog/Dnmt3b Pluripotency Screen

3 × 10^3^ mESCs were seeded in 96-well plates and 1 μM inhibitors applied for 48 hr. Cells were lysed, and clarified extract was transferred onto a nitrocellulose membrane using a 96-well vacuum dot blot manifold and immunoblotted for Nanog and Dnmt3b using Li-Cor 800nm anti-rabbit (Nanog) and anti-mouse-HRP (Dnmt3b), respectively.

### Erk5 Gene Sequencing

Genomic DNA was extracted using the DNeasy Blood and Tissue Kit (QIAGEN), and the Erk5 gene was analyzed by PCR sequencing (forward: 5′-AGCTGATCCCGACTGTGTCT-3′, reverse: 5′-CAGGTGGCCATCAAGAAGAT-3′).

### mESC Phenotyping

mESCs were cultured at 1,000 cells per six wells for 6–7 days prior to AP staining solution. For colony phenotyping, mESCs were plated at 200 cells per 10-cm dish and analyzed after 7 days. For proliferation assay, mESCs were seeded at 10,000 cells per six wells and counted.

### Embryoid Body Differentiation

EBs were formed by aggregating 60,000 mESCs/ml in the absence of LIF for 4 days before transfer to gelatin-coated plates for an additional 4 days.

### Fluorescence Activated Cell Sorting

At day 4, EBs were treated with VEGF (5 ng/ml; Peprotech), Activin A (4 ng/ml; R&D), and BMP4 (0.5 ng/ml; R&D) and dissociated at day 7 by incubation with TrypLE (Invitrogen) and stained.

### RNA Extraction and qPCR

RNA was extracted using the OMEGA total RNA kit and reverse transcribed using iScript reverse transcriptase (Bio-Rad). qPCR was performed using SsoFast EvaGreen Supermix (Bio-Rad). The ΔCt method using GAPDH as a reference gene was used to analyze relative expression and the 2-ΔΔCt (Livak) method used to normalize to control. Primers used are listed in Table S4.

### Erk5 Immunoprecipitation Kinase Assay

Erk5 was immunoprecipitated from mESC lysate using 5 μg anti-Erk5 antibody. Beads were washed three times in lysis buffer containing 0.5M NaCl, then resuspended in a total volume of 25 μl kinase assay buffer (50 mM Tris HCl [pH 7.5], 0.1 mM EGTA, 10 mM MgCl_2_, 2 mM DTT, and 0.1 mM [γ- ^32^P]-ATP [500 cpm/pmol]) and incubated at 30°C for 30 min. The assay was terminated by SDS sample buffer and heating and analyzed by SDS-PAGE and autoradiography.

### Recombinant ERK5 Kinase Assay

200 ng pure active ERK5 was incubated with the indicated inhibitor in 50 mM Tris-HCl (pH 7.5), 0.1 mM EGTA, and 1 mM 2-mercaptoethanol. The reaction was initiated by adding 10 mM magnesium acetate, 50 μM [γ-^32^P]-ATP (500 cpm/pmol), and 250 μM PIMtide (ARKKRRHPSGPPTA) and incubated at 30°C for 20 min. The assay was terminated by applying the reaction mixture onto p81 paper and incorporated radioactivity measured.

### AlphaScreen Brd4-1 Bromodomain Binding Assay

Brd4-1 binding assay was performed by Reaction Biology Corp. using His-tag Brd4-1 proteins expressed in *Escherichia coli* and biotinylated acetylated peptides. Brd4-1 protein and inhibitors were preincubated for 30 min in 50 mM HEPES (pH 7.5), 100 mM NaCl, 0.05% CHAPS, and 0.1% BSA, then incubated for a further 30 min after addition of tetra-acetylated histone H4 peptide (H4 (1-21) K5/8/12/16(Ac)4-Biotin) and the streptavidin-coated donor beads. Ni-chelate acceptor beads were added and incubated for 1 hr, and signals were measured by Envision (Ex/Em = 680/520–620 nm).

### Statistical Analysis

Data are presented as the average with error bars indicating SD. Statistical significance of differences between experimental groups was assessed using a Student’s t test. Differences in averages were considered significant if p < 0.05. Representative western blots are shown.

## Author Contributions

C.A.C.W., R.F.-A., J.W., and G.M.F. designed, performed, and analyzed experiments. R.T. and N.S.G. provided reagents and expertise. G.M.F. wrote the paper.

## Supplemental Information

Document S1. Supplemental Experimental Procedures, Figures S1–S3, and Tables S1–S4

Document S2. Article plus Supplemental Information
