# Supplementary material for: Erk5 Is a Key Regulator of Naive-Primed Transition and Embryonic Stem Cell Identity
Source: Cell Rep. 2016 Aug 4;16(7):1820–8. doi: 10.1016/j.celrep.2016.07.033 (PMC4987282; doi:10.1016/j.celrep.2016.07.033)
Supplement: Document S1. Supplemental Experimental Procedures, Figures S1–S3, and Tables S1–S4 [file mmc1.pdf]

Cell Reports, Volume *16*

Supplemental Information

Erk5 Is a Key Regulator of Naive-Primed Transition and Embryonic Stem Cell Identity

Charles A.C. Williams, Rosalia Fernandez-Alonso, Jinhua Wang, Rachel Toth, Nathanael S. Gray, and Greg M. Findlay

Supplemental Information

Supplemental Figure legends

Figure S1, related to Figure 2 A) Erk5+/+ mESCs were treated with XMD8-92 at varying concentrations for 2 days, and relative cell survival determined. Data are represented as average ± SD from 3 independent experiments. B) mESCs were treated with the indicated concentrations of AZD4547, Ruxolitinib, XMD8-92 and JWG-071 for 48h, and levels of Nanog, Dnmt3b and Erk1/2 determined by immunoblotting. Intervening lanes were removed, as indicated by a dotted line (n=3). C) mESCs were treated with 1μM AZD4547, Ruxolitinib and JWG-071 for 48h, and Dnmt3b mRNA levels determined by qRT-PCR. Data are represented as average ± SD from a representative experiment (n=3). D) mESCs were transfected with control non-targeting siRNA or siRNA targeting Dnmt3b. Dnmt3b and Sox2 were analysed by immunoblotting (n=3). E) mESCs were transfected with Cas9 D10A and either control gRNAs or gRNAs specifically targeting Brd4, Grb2 or Stat3 and levels of Brd4, Grb2, Stat3 and Erk1/2 determined by immunoblotting (n=3).

Figure S2, related to Figure 3. A) Erk5+/+ or Erk5-/- mESCs were transfected with either empty vector or Erk5 cDNA, and Klf2 mRNA (left panel) or Erk5 and Erk1/2 expression (right panel) determined by immunoblotting. Data are represented as average ± SD from a representative experiment (n=3). B) Erk5+/+ mESCs were treated with indicated concentrations of Mek5 inhibitor BIX 02189 or Erk5 inhibitor JWG-071. Klf2 and Erk5 levels were determined by immunoblotting (n=3). C) Erk5+/+ mESCs were transfected with empty vector or Mek5DD cDNA and Fgf5 mRNA levels at indicated days post transfection determined by qRT-PCR. The ratio of Mek5DD/Control is represented as average ± SD from 3 independent experiments. D) Erk5-/- mESCs were transfected with either empty vector or Erk5 cDNA, and Klf4, Erk5 and Erk1/2 expression determined by immunoblotting (n=3). E) Erk5+/+ or Erk5-/- mESCs were LIF deprived for the indicated time and Klf4, Erk5 and Erk1/2 expression determined by immunoblotting. Intervening lanes were removed, as indicated by a dotted line (n=3).

Figure S3, related to Figure 4. A) Representative images of alkaline phosphatase stained Erk5+/+ and Erk5-/- mESC colonies B) Erk5+/+, Erk5ΔN/- and Erk5 -/- mESCs were counted over 5 days to determine relative proliferation rate (n=3). C) A single Erk5-/- mESC clone was used to generate clones of Erk5-/- mESCs re-expressing Erk5, and percentage beating compared to parental Erk5-/- and Erk5ΔN/- mESC lines. Data are represented as average ± SD for 4 independent clones. D) Representative images of Erk5+/+ and Erk5-/- embryoid bodies.

Figure S1, related to Figure 2

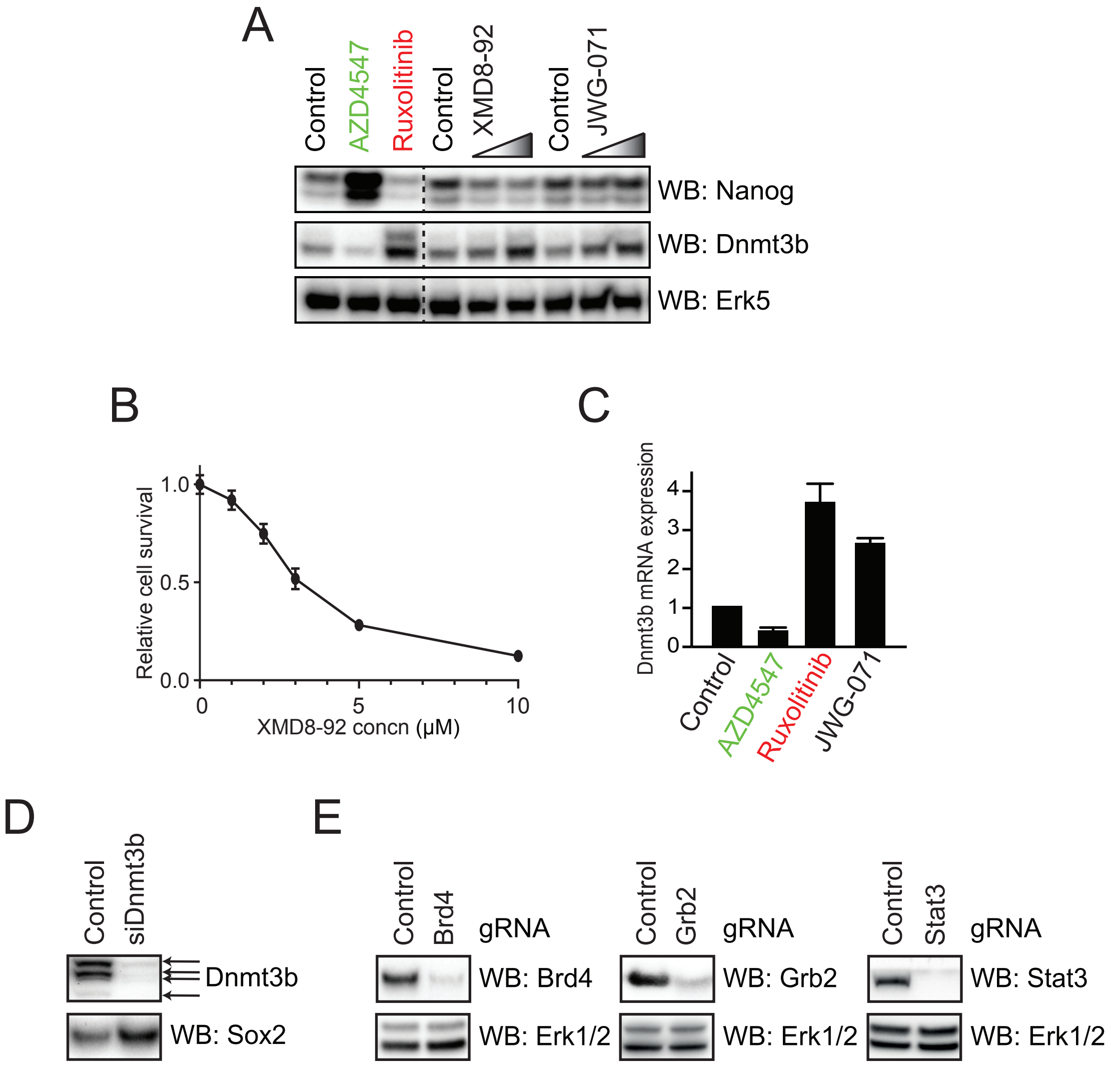

Figure S2, related to Figure 3

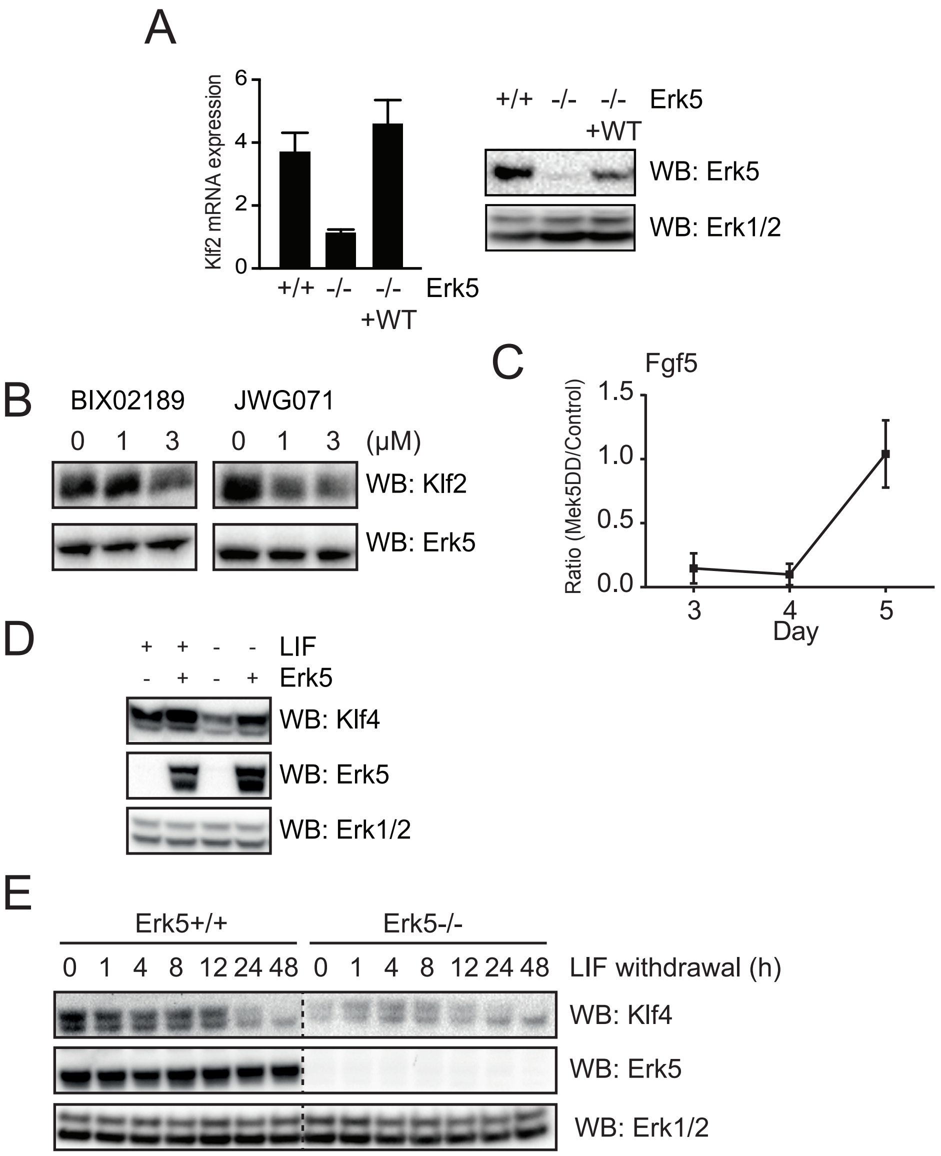

Figure S3, related to Figure 4

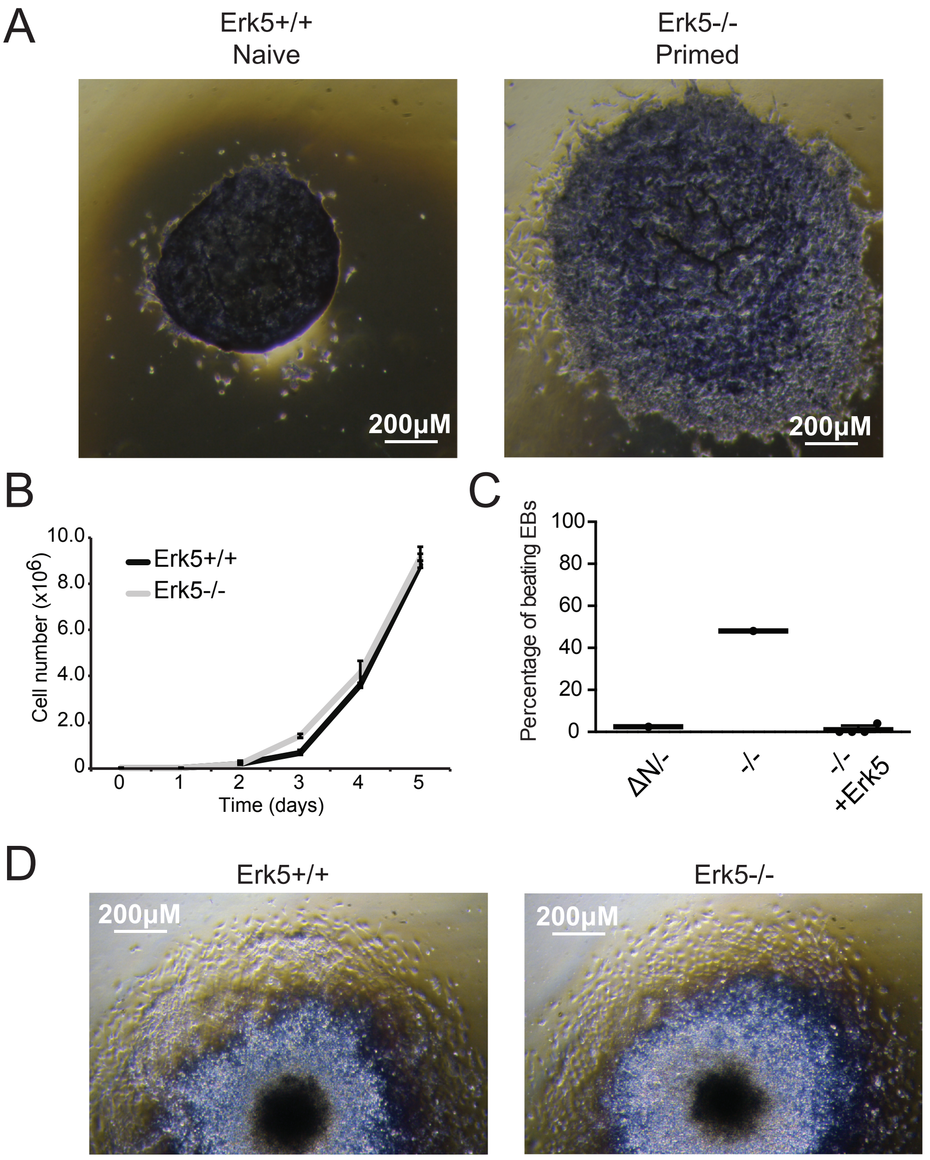

Supplemental Tables

Table S1. Full list of primed hits from screen, related to Figure 1

Primed Hits (<0.5 Nanog:Dnmt3b)

**Inhibitor**	**Primary Target(s)**	**Comments**
Ruxolitinib	JAK	Known pluripotency regulator
GDC-0879	B-Raf	Known pluripotency regulator
17-AAG	Hsp90	Broad spectrum kinase inhibitor
BX795	TBK1/PDK1	Known pluripotency regulator
TPCA-1	IKK2, STAT3	Known pluripotency regulator
VX-745	p38 MAPK	Known pluripotency regulator
OSI-930	c-Kit, VEGFR2	Broad spectrum kinase inhibitor
XL765 (SAR245409)	mTOR/PI3K	Known pluripotency regulator
D4476	CK1, ALK5 (TGFβRI)	Known pluripotency regulator
KIN001-269	FMS	Broad spectrum kinase inhibitor
LDN193189	ALK2 (BMPR)	Known pluripotency regulator
SB203580	p38 MAPK	Known pluripotency regulator
ABT-869 (Linifanib/AL-39324)	FLT-3	Broad spectrum kinase inhibitor
KIN001-236	Tie2	Broad spectrum kinase inhibitor
PIK-93	PI3K, PI4KIIIβ	Known pluripotency regulator
A769662	AMPK	Known pluripotency regulator
TGX221	PI3K	Known pluripotency regulator
SU6656	Src	Broad spectrum kinase inhibitor
BS-181	CDK7	Broad spectrum kinase inhibitor
KIN001-260 (Bayer IKKβ inhibitor)	IKKβ	Known pluripotency regulator
**XMD8-85**	**ERK5**	
Brivanib	VEGFR, PDGFR	Broad spectrum kinase inhibitor
CC-401	JNK	Known pluripotency regulator
CYT387	JAK	Known pluripotency regulator
NVP-BHG712	EPHB4	Broad spectrum kinase inhibitor
ABT-737	Bcl-2	Non-kinase
Regorafenib (BAY 73-4506)	c-KIT, VEGFR2, B-Raf	Broad spectrum kinase inhibitor
BAY-439006	B-Raf	Known pluripotency regulator
RAF-265 (CHIR-265)	B-Raf	Known pluripotency regulator
BIRB-796 (Doramapimod)	p38 MAPK	Known pluripotency regulator

Table S2. Full list of naïve hits from screen, related to Figure 1

Naïve Hits (>2 Nanog:Dnmt3b)

**Inhibitor**	**Primary Target(s)**	**Comments**
AZD8330	MEK1/2	Known pluripotency regulator
GSK-1120212 (JTP-74057)	MEK1/2	Known pluripotency regulator
BIBF-1120 (Vargatef)	VEGFR, PDGFR and FGFR	Known pluripotency regulator
WZ-4-145	CSF1R/DDR1/EGFR/TIE1/ PDGFR2	Broad spectrum kinase inhibitor
PD173074	FGFR	Known pluripotency regulator
HG-6-64-01	Tyrosine kinases	Broad spectrum kinase inhibitor
WZ-7043	CSF1R/DDR1/FGFR/TAO1	Known pluripotency regulator
AP24534 (Ponatinib)	BCR-ABL	Broad spectrum kinase inhibitor
**NU7441 (KU 57788)**	**DNA-PK**	**Under investigation**
AZD4547	FGFR	Known pluripotency regulator
KIN001-043	GSK3	Known pluripotency regulator
BGJ398	FGFR	Known pluripotency regulator
HG-14-10-04	ALK	Broad spectrum kinase inhibitor
GW786034 (Pazopanib)	VEGFR1	Broad spectrum kinase inhibitor
AZD6244 (Selumetinib)	MEK1/2	Known pluripotency regulator
MK2206	AKT1	Known pluripotency regulator
GDC-0941	PI3K	Known pluripotency regulator
R406	Syk	Broad spectrum kinase inhibitor
AZD0530 (Saracatinib)	Src	Broad spectrum kinase inhibitor
PD0325901	MEK1/2	Known pluripotency regulator
HG-5-113-01	LOK/LTK/TRCB/ABL(T315I)	Broad spectrum kinase inhibitor
Dasatinib	Src family	Broad spectrum kinase inhibitor
AZD6482	PI3K	Known pluripotency regulator

Table S3. Sequences of Erk5 gene targeted mESC lines, related to Figure 2.

Erk5 Line	Allele Summary	DNA Sequence
+/+	wild-type	ACCAATGCCAAACGGACCCTCAGGGAGCTGAAGATCCTCAAACACTTCAAACACGACAATATCATCGCCATCAAGGACATCCTGAAGCCTACTGTG
ΔN/-	A1 in frame	ACCAATGCCAAACGGACCCTCAGGGAGCTGAAGATCCTCAAAC AGGACATCCTGAAGCCTACTGTG
	A2 large insertion	
ΔN/-	A1 in frame	ACCAATGCCAAACGGACCCTCAGGGAGC---------------------AACACGACAATATCATCGCCATCAAGGACATCCTGAAGCCTACTGTG
	A2 frameshift	ACCAATGCCA--AAGGACATCCTGAAGCCTACTGTG
-/-	A1 frameshift	ACCAATGCCAAACGGACCCTCAGGGAGCTGAAGATCCTCAAACACT--ACTGTG
	A2 frameshift	ACCAATGCCAAACGGACCCTCAGGGAGCTGAAGATCCTCAAAC---ACTGTG
ΔN/-	A1 in frame	ACCAATGCCAAACGGACCCTCAGGGAGCTGAAGATCCTCAAAC------------------------------AGGACATCCTGAAGCCTACTGTG
	A2 large insertion	
-/-	A1 frameshift	ACCAATGCCAAACGGACCCTCAGGGAGCTGAAGAT-----------------------------TCGCCATCAAGGACATCCTGAAGCCTACTGTG
	A2 frameshift	ACCAATGCCAAACGGACCCTCAGGGAGC-------------------------------------CGCCATCAAGGACATCCTGAAGCCTACTGTG
	A3 frameshift	ACCAATGCCAAACGGACCCTCAGGGAG--------------ACACTTCAAACACGACAATATCATCGCCATCAAGGACATCCTGAAGCCTACTGTG
-/-	A1 frameshift	ACCAATGCCAAACGGACCCTCAGGG------------------TTTGAGGACACGACAATATCATCGCCATCAAGGACATCCTGAAGCCTACTGTG
	A2 large insertion	

N.B. large insertions were not sequenced

Supplemental Experimental Procedures

Table S4. Primers used for qRT-PCR, related to Experimental Procedures

Gene	Forward	Reverse
Nanog	CTCATCAATGCCTGCAGTTTTTCA	CTCCTCAGGGCCCTTGTCAGC
Oct4	AGCTGCTGAAGCAGAAGAGG	AGATGGTGGTCTGGCTGAAC
Klf2	CTCAGCGAGCCTATCTTGCC	CACGTTGTTTAGGTCCTCATCC
Rex1	CCCTCGACAGACTGACCCTAA	TCGGGGCTAATCTCACTTTCAT
Essrb	AACCGAATGTCGTCCGAAGAC	GTGGCTGAGGGCATCAATG
Fgf5	GCTGTGTCTCAGGGGATTGT	CACTCTCGGCCTGTCTTTTC
Brachyury	TCCCGAGACCCAGTTCATAG	TTCTTTGGCATCAAGGAAGG
Pdgfra	TCCATGCTAGACTCAGAAAGTCAA	TCCCGGTGGACACAATTTTTC
Flk1	TCCAGAATCCTCTTCCATGC	AAACCTCCTGCAAGCAAATG
GAPDH	CTCGTCCCGTAGACAAAA	TGAATTTGCCGTGAGTGG
Sox1	TTCCCCAGGACTCCGAGGCG	GCTGTGTGCCTCCTCTGCGG
Nkx2-5	ACCTTTAGGAGAAGGGCGATGACT	AAGTGGGATGGATCGGAGAAAGGT
Nppa	CAGAATCGACTGCCTTTTCC	GGGGGTAGGATTGACAGGAT
Tnt	ACCCTCAGGCTCAGGTTCA	GTGTGCAGTCCCTGTTCAGA

Supplemental References

Bourillot P-Y, Aksoy I, Schreiber V, Wianny F, Schulz H, Hummel O, Hubner N, Savatier P (2009) Novel STAT3 target genes exert distinct roles in the inhibition of mesoderm and endoderm differentiation in cooperation with Nanog. Stem cells (Dayton, Ohio) 27**:** 1760-1771

Ceccaldi R, Parmar K, Mouly E, Delord M, Kim JM, Regairaz M, Pla M, Vasquez N, Zhang Q-S, Pondarre C et al (2012) Bone marrow failure in Fanconi anemia is triggered by an exacerbated p53/p21 DNA damage response that impairs hematopoietic stem and progenitor cells. Cell stem cell 11**:** 36-49

Chazaud C, Yamanaka Y, Pawson T, Rossant J (2006) Early lineage segregation between epiblast and primitive endoderm in mouse blastocysts through the Grb2-MAPK pathway. Developmental cell 10**:** 615-624

Ciceri P, Müller S, O'Mahony A, Fedorov O, Filippakopoulos P, Hunt JP, Lasater EA, Pallares G, Picaud S, Wells C et al (2014) Dual kinase-bromodomain inhibitors for rationally designed polypharmacology. Nature chemical biology 10**:** 305-312

Davies SP, Reddy H, Caivano M, Cohen P (2000) Specificity and mechanism of action of some commonly used protein kinase inhibitors. The Biochemical journal 351**:** 95-105

Di Micco R, Fontanals-Cirera B, Low V, Ntziachristos P, Yuen SK, Lovell CD, Dolgalev I, Yonekubo Y, Zhang G, Rusinova E et al (2014) Control of embryonic stem cell identity by BRD4-dependent transcriptional elongation of super-enhancer-associated pluripotency genes. Cell reports 9**:** 234-247

Feldman B, Poueymirou W, Papaioannou V, DeChiara T, Goldfarb M (1995) Requirement of FGF-4 for postimplantation mouse development. Science 267**:** 246-249

Hayashi M, Kim S-W, Imanaka-Yoshida K, Yoshida T, Abel ED, Eliceiri B, Yang Y, Ulevitch RJ, Lee J-D (2004) Targeted deletion of BMK1/ERK5 in adult mice perturbs vascular integrity and leads to endothelial failure. The Journal of clinical investigation 113**:** 1138-1148

Heinrich PC, Behrmann I, Müller-Newen G, Schaper F, Graeve L (1998) Interleukin-6-type cytokine signalling through the gp130/Jak/STAT pathway. The Biochemical journal 334 (Pt 2**:** 297-314

Hirasawa R, Sasaki H (2009) Dynamic transition of Dnmt3b expression in mouse pre- and early post-implantation embryos. Gene expression patterns : GEP 9**:** 27-30

Honda T, Obara Y, Yamauchi A, Couvillon AD, Mason JJ, Ishii K, Nakahata N (2015) Phosphorylation of ERK5 on Thr732 is associated with ERK5 nuclear localization and ERK5-dependent transcription. PloS one 10**:** e0117914

Kasler HG, Victoria J, Duramad O, Winoto A (2000) ERK5 is a novel type of mitogen-activated protein kinase containing a transcriptional activation domain. Molecular and cellular biology 20**:** 8382-8389

Kunath T, Saba-El-Leil MK, Almousailleakh M, Wray J, Meloche S, Smith A (2007) FGF stimulation of the Erk1/2 signalling cascade triggers transition of pluripotent embryonic stem cells from self-renewal to lineage commitment. Development 134**:** 2895-2902

Lacout C, Pisani DF, Tulliez M, Gachelin FM, Vainchenker W, Villeval J-L (2006) JAK2V617F expression in murine hematopoietic cells leads to MPD mimicking human PV with secondary myelofibrosis. Blood 108**:** 1652-1660

Lang SH, Frame FM, Collins AT (2009) Prostate cancer stem cells. The Journal of pathology 217**:** 299-306

McKay R (1997) Stem Cells in the Central Nervous System. Science 276**:** 66-71

Muller S, Filippakopoulos P, Knapp S (2011) Bromodomains as therapeutic targets. In Expert Reviews in Molecular Medicine.

Nan J, Du Y, Chen X, Bai Q, Wang Y, Zhang X, Zhu N, Zhang J, Hou J, Wang Q et al (2014) TPCA-1 is a direct dual inhibitor of STAT3 and NF-κB and regresses mutant EGFR-associated human non-small cell lung cancers. Molecular cancer therapeutics 13**:** 617-629

Noble MEM, Endicott JA, Johnson LN (2004) Protein kinase inhibitors: insights into drug design from structure. Science (New York, NY) 303**:** 1800-1805

Singh SK, Clarke ID, Terasaki M, Bonn VE, Hawkins C, Squire J, Dirks PB (2003) Identification of a Cancer Stem Cell in Human Brain Tumors. Cancer Res 63**:** 5821-5828

Slack JM (2000) Stem cells in epithelial tissues. Science (New York, NY) 287**:** 1431-1433

Sohn SJ, Li D, Lee LK, Winoto A (2005) Transcriptional regulation of tissue-specific genes by the ERK5 mitogen-activated protein kinase. Molecular and cellular biology 25**:** 8553-8566

Suzaki Y, Yoshizumi M, Kagami S, Koyama AH, Taketani Y, Houchi H, Tsuchiya K, Takeda E, Tamaki T (2002) Hydrogen peroxide stimulates c-Src-mediated big mitogen-activated protein kinase 1 (BMK1) and the MEF2C signaling pathway in PC12 cells: potential role in cell survival following oxidative insults. The Journal of biological chemistry 277**:** 9614-9621

Veraksa A, Del Campo M, McGinnis W (2000) Developmental patterning genes and their conserved functions: from model organisms to humans. Molecular genetics and metabolism 69**:** 85-100

Yeo J-C, Jiang J, Tan Z-Y, Yim G-R, Ng J-H, Göke J, Kraus P, Liang H, Gonzales KAU, Chong H-C et al (2014) Klf2 is an essential factor that sustains ground state pluripotency. Cell stem cell 14**:** 864-872
